# Transmission Dynamics of Urogenital Schistosomiasis in the Rural Community of Ebonyi State, South Eastern Nigeria

**DOI:** 10.1155/2019/7596069

**Published:** 2019-01-01

**Authors:** F. N. Afiukwa, D. E. Nwele, O. E. Uguru, G. A. Ibiam, C. S. Onwe, A. U. Ikpo, N. B. Agumah, O. F. Odoemena

**Affiliations:** ^1^Department of Applied Microbiology, Faculty of Science, Ebonyi State University Abakaliki, Nigeria; ^2^Department of Applied Biology, Faculty of Science, Ebonyi State University Abakaliki, Nigeria; ^3^Department of Medical Microbiology, Faculty of Basic Medicine, Ebonyi State University Abakaliki, Nigeria; ^4^Department of Medical Laboratory Sciences, Faculty of Health Sciences Ebonyi State University Abakaliki, Nigeria; ^5^Department of Parasitology and Entomology, Nnamdi Azikiwe University Awka, Nigeria; ^6^Department of Human Anatomy, Faculty of Basic Medicine, Ebonyi State University Abakaliki, Nigeria

## Abstract

This study accessed the dynamics of urogenital schistosomiasis transmission in Nkalagu Community. A total of 500 mid-day urine samples were collected and transported to Microbiology Laboratory, Ebonyi State University, for analysis. 10ml each of the urine samples was centrifuged at 2500 rpm for 5 minutes. Transmission potential of snail intermediate host of Schistosomes collected from different sampling station at the transmission sites within the study community was equally accessed. The snail species collected were placed individually into a clean beaker with little quantity of water and then subjected to shedding light for 2 hours. Data obtained were entered in excel spread sheet and analyzed using chi square test. The result obtained shows that 205 (41%) out of 500 individuals examined were excreting* S. haematobium* ova in their urine. The highest prevalence of infection (23%) was found among 11-20-year age groups. Males were more infected (25.4%) than their female counterparts (15.6%), although this was not statistically significant (p > 0.05). A total of 283 snails belonging to two Bulinus species (*B. globosus* and* B. truncatus*) were collected from the four sites sampled.* Bulinus globosus* recorded the highest species abundance (177) with the highest occurrence in site A. 52 (18.4%) out of 283 snails collected were infected with cercariae, and the highest cercariae infection (12.0%) was recorded among* B. globosus*. With prevalence of 41% among the human population and the prevalence of 18.4% patent infection among the snail intermediate hosts, urogenital schistosomiasis is still a public health problem in the study area and falls within the WHO classification of endemic area. Public health campaign is recommended in order to educate the people on the mode of transmission and control of the disease.

## 1. Introduction

Urinary schistosomiasis is a parasitic disease caused by the blood fluke of the genus* Schistosoma*. The disease is endemic in 76 countries in the Middle East and most of the African countries [1]. It is one of the major public health problems facing humanity, with severe and economic consequences [2]. It is the most prevalent of waterborne diseases [3]. Human waste in water containing intermediate hosts is the single most important epidemiological factor in schistosomiasis transmission as well as the availability of suitable snail host [4]. The transmission of schistosomiasis is associated with water development projects such as dams for irrigation systems and fish-farming, as the snail intermediate hosts of the parasites breed in them and human water contact also takes place in such water body [5]. Urinary schistosomiasis being a water-based disease is spread through contact with water in which snail habouring and shedding the infective stage (cercariae) of the parasite (schistosome) are present [6, 7]. As with other zoonotic infections, urinary schistosomiasis has a natural habitat in a well-defined ecosystem. The parasite, intermediate hosts, and the human host form an association within which the parasite reproduces and is disseminated [8].

Nigeria is one of the countries known to be highly endemic for urinary schistosomiasis with more than 100 million people at risk and about 25 million already infected [1, 9]. School aged children are at high risk of infection and prevalence usually peaks between the ages of 8 and 15 years [10–12]. As a result of low level of resistance and intensive water contact when playing and swimming, children aged between 10 and 15 years are the most heavily infected [7, 13]. Increased population movements help to spread the disease, and* Schistosomiasis* is now occurring increasingly in peri-urban areas in endemic countries [14].

Typical signs of urinary schistosomiasis are macro- and microhaematuria, proteinuria and leukocyturia [11, 15] and the risk of haematuria, dysuria, nutritional deficiencies, lesion of the bladder, kidney failure, and child growth retardation are well established [16].

Human infection by* Schistosomes* frequently occur in stable foci and is dependent on snail infection and contact patterns of humans with water infested with cercariae [17]. The risk of infection in these transmission sites may be routinely estimated by detecting infected snails capable of shedding cercariae. However, since seasonal fluctuations exist in snail population densities, infection rates, and cercarial output, information on both snail infection and presence and distribution of cercariae is required for evaluating the risk of infection. Information on presence of cercariae in water is particularly important when only one of many snails is infected yet capable of shedding enough cercariae to maintain high endemicity [18].

## 2. Study Area and Population

The study was carried out for a period of 12 months in Nkalagu Community, a* Schistosomiasis *endemic area in Ebonyi State. Nkalagu Community is made up of five (5) villages: Ishiagu, Amanvu, Uwule, Imoha, and Akiyi. The geographical coordinate of the study area lies between longitude 54°E and latitude 60°N. The climate of the study area is tropical with mean daily temperature of 30 ± 5°C for the most of the year. The annual rainfall is between 1900 and 2200mm, with wet and dry seasons. The vegetation is typically rainforest. Several freshwater habitats intersect the study area, some of which include ponds, streams, dams, and rivers. These water bodies form the major source of water supply to the residents of the study area. During both wet and dry seasons, activities increase around these water bodies as people converge to use them for domestic, agricultural and recreational activities all of which could predispose them to urinary schistosomiasis. The study area was selected based on previous report on schistosomiasis prevalence as well as laboratory/confirmatory evidence of the disease in the area during preliminary survey. All individuals resident in the study area for the past one year before the study were included for participation.

## 3. Ethical Consideration and Consent

Ethical clearance was obtained from Ebonyi State Ministry of Health who issued a letter permitting the study to the community leaders. The study participants were informed about the purpose, procedures, and potential risk and benefits of the study and were invited to approve their participation in the study. A written informed consent was signed by each study participant at the beginning of the study.

## 4. Study Population and Design

This present study includes 500 individuals of different age groups and sex who consented to participate in the study. The participants were selected using random sampling technique on a house to house basis as described by Nwosu* et al.* [19].

A 20ml of clean catch, midstream urine samples was collected in autoclaved wide mouthed, leak proof universal containers by the study participants themselves as described by WHO [20] and Uneke* et al.* [9]. Samples were collected between 10.00 am and 14.00 hours, labeled with unique identifiers and transferred to the laboratory in a cold box with ice packs. The urine samples were processed 1-2 hours after collection.

## 5. Laboratory Analysis of Sample

In the laboratory, 10ml terminal urine was centrifuged at 2,500 revolution per minute (rpm) for 5 minutes in order to concentrate eggs of schistosome as described by Cheesbrough [21]. The urine sedimentation technique described was used to detect the presence of* S. haematobium* ova in the urine samples mounted under microscope. Intensity of the infection in each case was reported as the number of ova/10ml of urine and was categorized as light infection (<50 ova/10ml of urine) or heavy infection (>50 ova/10ml of urine) [21].

## 6. Snail Sampling Technique/Procedure

The sampled rivers (Abashi and Edenvu) for this study were divided into different sampling stations (A, B, C, and D) on the basis of accessibility and variations in site ecology. Each of the sampling stations was sampled on monthly basis for two seasons (wet and dry seasons).

Two major techniques were adopted for the snail sampling; the scooping technique and the manual hand picking technique. Scooping net technique involves the use of scooping net in collecting the snails. The scoop net was lowered under the vegetation at the bank of the river and scooped in order to catch the snails attached to the shrubs as described by Oguoma* et al.* [22] and Ivoke* et al.* [8]. This was repeated severally at random in different locations on each day of sampling. In manual hand picking, long forceps were used to pick snails resting on vegetation and under the stream. Snails were also picked with hands covered with gloves to avoid direct contact with infested water and snails as described by Oguoma* et al.* [22]. All snails collected each day of sampling at each station were kept in a prelabeled transparent bucket containing water from the site. The sample was transported to the laboratory for identification and examination using key provided by Brown, [23] and Okafor and Obiezue, [24]. The identified snail species was later confirmed by Senior Parasitologist in the Department of Applied Biology of the Ebonyi State University.

## 7. Laboratory Examination of Snail for Cercariae Shedding

In the laboratory, the collected snails were placed individually into a clean 100ml beaker with little quantity of clean water. Each beaker was then placed under a 100 watt bulb (shedding light), as described by Okafor [25] and Ivoke* et al.* [8] to stimulate the emergence of the cercaria if present in the snail. The beaker was allowed to stay under the shedding light for about 2hrs after which the snail was transferred to another beaker and the sheded cercaria counted. The process was repeated until no cercaria was coming out of the snail again. Adequate care was taken during this process not to overheat and kill the snails. In the absence of shedding light, direct sunlight was used to achieve the same purpose. Beakers containing snail and water were placed directly under the sun and monitored for the emergence of cercaria as described by Okoli and Iwuala [26]. All cercaria encountered during the process was counted and recorded.

## 8. Data Analysis

Data collected was double entered in Microsoft excel and checked for consistency and analyzed using SPSS version 20.0. The* S. haematobium *infection prevalence, defined as the percentage of individual with* S. haematobium *eggs in urine, was calculated. Chi square test was used to determine statistical difference between two variables.

## 9. Results

**Figure 1:**
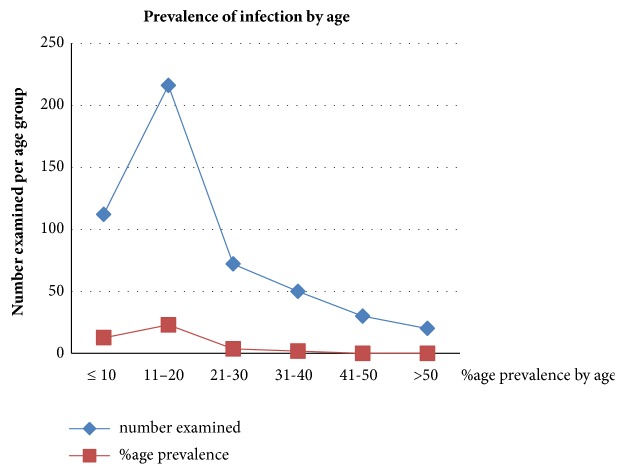
Prevalence of urogenital schistosomiasis by age.

A total of 500 participants, comprising both males and females, were included in this study. The overall infection prevalence was 41% with the highest infection prevalence (23%) occurring among age group 11-20 years and was closely followed by ≤10 years age group (12.6%). The least infection prevalence (1.8%) was recorded among 31-40 years age groups. Statistically there was no significance difference in the prevalence of infection among the age groups (X^2^= 1.02; df = 5, P> 0.05) (Figure 1). See Figure 3 for the image of the* S. haematobium* ova recovered from the urine sample.

**Figure 2:**
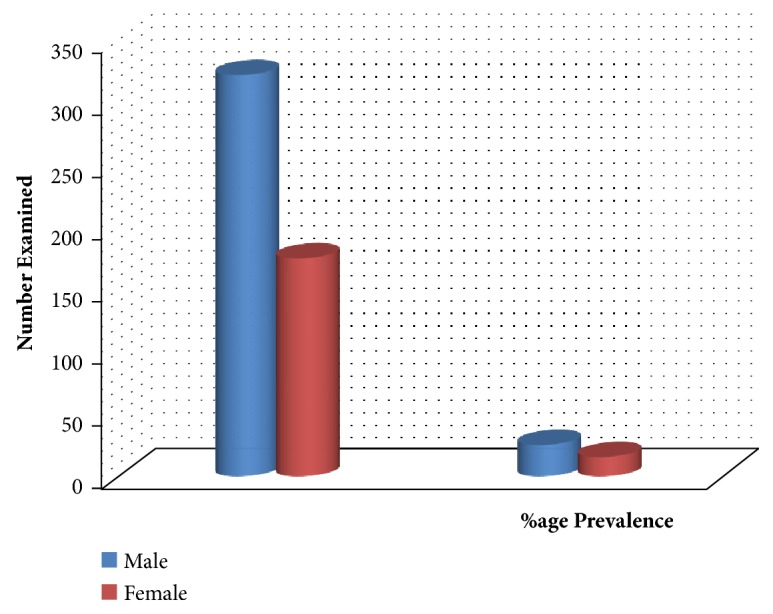
Prevalence of infection by sex.

**Figure 3:**
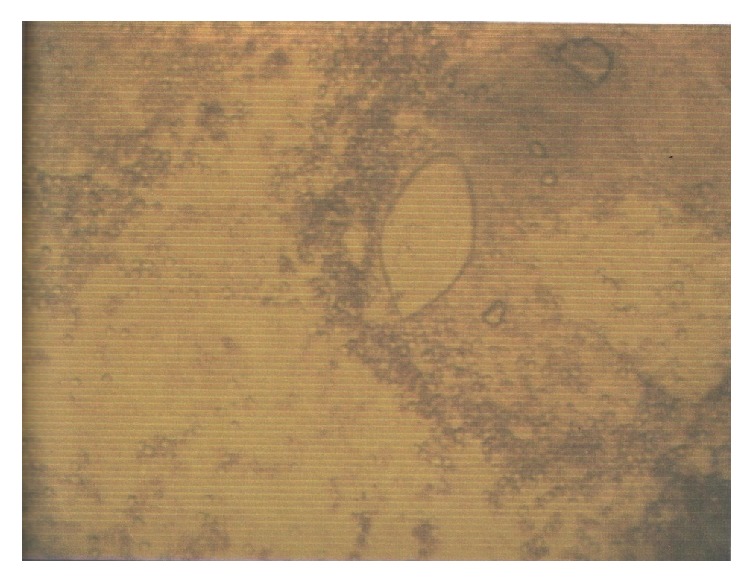
Ova of* S. haematobium *recovered from urine sample.

The result obtained on the distribution of the infection by sex showed that the overall prevalence of infection was highest among males (25.4%) than their female counterparts (15.6%). However, there was no significant difference in the prevalence in both sexes (P> 0.05) (Figure 2).

**Figure 4:**
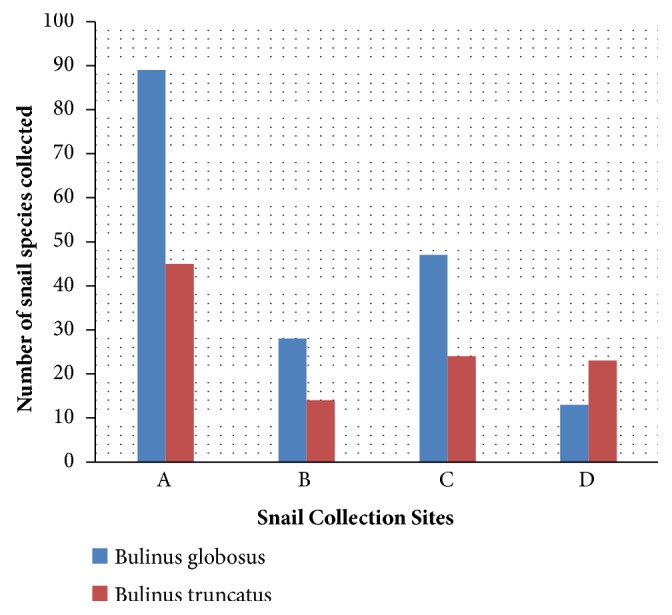
Distribution of different snail species in different sites sampled.

A survey of the abundance and species distribution of the snail intermediate host of urogenital* Schistosomiasis *around the fresh water bodies in the study community showed a total snail population of 283 snails belonging to two species (*Bulinus globosus *and* Bulinus truncatus*). The highest snail population was recorded among* B. globosus* (Figure 4).

The distribution of snail species in the different stations sampled varied considerably with the site and months of the survey. The highest number of snail species was encountered in site A (134), while site D recorded the least number of snail (36). Monthly abundance also shows that more snails were collected in the months of June, August, and October when water current capable of dislodging snails was reduced while least snail number (6, 10 and 18) was found between February, March, and April, respectively, due to a decrease in the volume of the river. No snail was collected in the month of January (Table 1).

**Figure 5:**
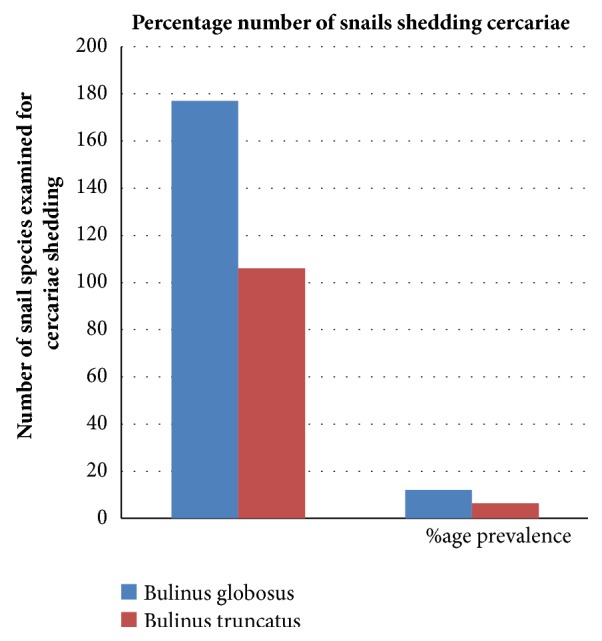
Percentage output of cercariae shedding by different snail species.

**Figure 6:**
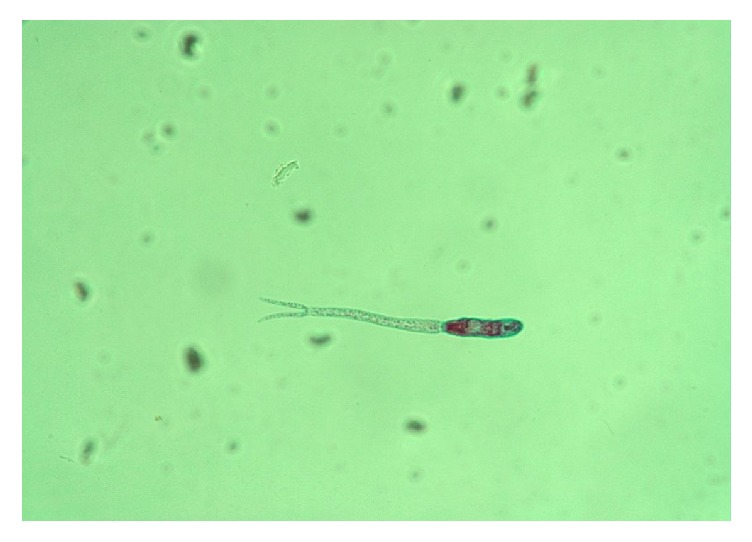
Image of cercariae taken from the microscope.

Figure 5 shows the percentage output of cercariae shedding by different snail species when subjected to shedding light. Out of the 283 snails collected and subjected to shedding light, only 52, (18.4%) were shedding cercariae with the highest number of snails shedding cercariae, recorded among* Bulinus globosus,* 34(12.0%) while the least number of snail shedding cercariae was found among* B. truncatus,* 18(6.4%). The image of the cercariae captured from the microscope is show in Figure 6.

## 10. Discussion

Studies have shown that urinary Schistosomiasis is a major health problem in the rural areas of the Middle East and most African countries [20]. It remains one of the major health problems facing developing countries. The endemicity of the disease in many rural areas was attributed to poor living conditions, ignorance, inadequate sanitation, inadequate water supply, personal and environmental hygiene, and water contact activity with snail infected river, streams, and ponds [20].

This study recorded* Schistosoma haematobium* prevalence of 41% in the community studied. The result suggests that the study area falls within the WHO classification as endemic area [2]. The result is consistent with many other studies in Ebonyi State which shows that the disease is endemic in many rural communities of the state [9, 19, 27, 28]. However, the prevalence recorded in this present study is higher than the 22.1% reported by Anosike* et al.* [28] in the rural communities of Ebonyi State. It was equally higher than the 17.5% reported by Nwosu* et al.* [29] in Ezza North Local Government Area of Ebonyi State and was slightly lower than the 47.9% reported in Ohaukwu Local Government Area by Uneke* et al.* [9] in 2006. The major factors that contribute to high prevalence of urogenital schistosomiasis in any rural community are poor environmental sanitation, inadequate and indiscriminate disposal of human excreta (especially around freshwater bodies), and behavioural activities that brings people in contact with infested water bodies where transmission occurs. Uneke* et al.* [9] reported that low literacy, lack of basic amenities, inadequate and indiscriminate disposal of human sewage, and high water contact activity with snail infested pond, river, and stream may have been responsible for high endemicity of urinary schistosomiasis particularly in Ohaukwu.

The age prevalence of urogenital schistosomiasis as recorded in this study showed variation among different age groups studied with age group 11-20 years, recording the highest infection prevalence of 23%, and was followed by ≤10 years age groups with the prevalence of 12.6%. The high prevalence among 11-20 age groups indicates that they are the most active age group frequently engaging in activities that brings them in contact with infested water bodies which are possible transmission sites. This finding agrees with the earlier works of Okpala* et al.* [13] who equally reported highest prevalence among 11-15 age groups. Similarly, Okoli and Odaibo [30] reported school aged children especially those within the age group 11-15 as having the highest prevalence of* S. haematobium* infection. Nwosu* et al.* [19] equally reported highest prevalence among age group 11-20 years. The result of this study and other reports therefore indicate that transmission is usually high among children which are mainly due to their role in contamination of aquatic environment. According to Anosike* et al.* [31], children are the most infected group of people in endemic areas, thus contributing significantly to the potential contamination of the aquatic environment. The first two decades of life have been identified as the age of active life; hence increased human activity increases predisposition of people in this age group to infection especially through increased water contact activities [19]. The prevalence of infection recorded among ≤10 years age group could be due to early exposure to contaminated water as earlier reported by Nwosu* et al.* [19],

Regarding the prevalence of infection in relation to sex, this study showed that males were more infected with 25.4% prevalence than their female counterparts (15.6%). The high prevalence among males may be attributed to their behavioural practices which bring them in contact with the infested water than their female counterparts who usually engage more in domestic activities. Similar observations have been reported by Uneke* et al.* [9], Nwosu* et al.* [19], and Nwele* et al.* [7] in their separate studies. However, other studies reported that sex related prevalence is significant but could differ due to some variations in behavioural practices regarding water contact [32].

Survey of freshwater snail intermediate hosts around the river where transmissions takes place identified two snail species of* Bulinus (B. globosus and B. truncatus). *All snails recorded in this study have been previously reported in other parts of Nigeria [33].

There was considerable variation in the abundance of the snail species within the months of study. The highest number of snails was collected in the months of June, August, October, and May while the least number was seen in the month of February. The relative abundance of snails from May to October could be attributed to the availability of food which increases the growth of aquatic plants as well as the stability in water velocity. A drop in the snail population in the months of July and September indicates an increase in amount of rainfall with consequent increase in water current which is capable of sweeping snail population. Ndifon* et al.* [34], in his study, identified rainfall as the major factor influencing the availability of aquatic snails.

The snail population recorded in this study varied greatly with the different sites sampled with site A recording the highest snail abundance. The low number of snail recorded in sites B and D may have been occasioned by rainfall through flooding and subsequent flush out of snails from their habitats or by the rapid elevation of water levels resulting in the snails remaining submerged in the rivers. Several researchers in different part of the world have been able to demonstrate the usefulness of rainfall in increase or decrease of snail populations [33, 35–37].

The overall percentage of snail species shedding cercariae stood at 18.4% and this was lower than that reported by Ayanda [38], who recorded overall cercariae prevalence of 35.7%. The cercarial shedding was highest among* B. globosum *with prevalence of 12.0% while* B*.* truncatus* recorded the least prevalence of 6.4%. This, however, did not agree with the earlier report of Ayanda [38], who recovered 35.7% cercaria all from* B. globosus *from the stream of samara, in Zaria. The recovering of cercariae from the snail species collected is an indication that transmission is taking place in the study area and many individuals in these communities are not aware of the dangers associated with contact to these water bodies and therefore could be at greatest risk of infection. Though there was no particular trend in the variation of prevalence, it could be said from the result that increase in infected snail populations may result in an increase in the infection of human host by* Schistosome* cercariae resulting from their behavioural practices capable of bringing them in contact with the infested water. Although mortality due to schistosomiasis may be low, the disease imposes a heavy burden upon the health and wellbeing of individuals [39, 40].

## 11. Conclusion

The occurrence of cercaria in the collected snails indicates the presence of urinary schistosomiasis in the study area and this equally indicates that people making contact with the water where these snails were collected could be at risk of infection. Efforts should be geared towards educating the people of this community on the possible health risk involved when exposing their body to the river, as well as controlling the snail intermediate host.

## Figures and Tables

**Table 1:** Monthly abundance of *Bulinus* species in various sites sampled.

**Sampling months**	**No. of snail species**	**No. per sampling sites**				
		**A**	**B**	**C**	**D**	**Total**
**Jan.**	0	0	0	0	0	0
**Feb.**	6	6	0	0	0	6
**Mar.**	10	5	1	3	1	10
**Apr.**	18	7	5	4	2	18
**May**	40	17	8	10	5	40
**Jun.**	54	21	9	19	5	54
**Jul.**	28	16	2	7	3	28
**Aug.**	51	28	6	17	0	51
**Sept.**	25	16	0	9	0	16
**Oct.**	50	18	11	1	20	50

**Total**	283	134	42	71	36	283

## Data Availability

No data were used to support this study.
